# Clinical benefits and adverse effects of siwak (*S. persica*) use on periodontal health: a scoping review of literature

**DOI:** 10.1186/s12903-021-01950-0

**Published:** 2021-12-03

**Authors:** Haslinda Ramli, Tuti Ningseh Mohd-Dom, Shahida Mohd-Said

**Affiliations:** 1 Department of Family Oral Health, Faculty of Dentistry, Universiti Kebangsaan Malaysia, Jalan Raja Muda Abdul Aziz, 50300 Kuala Lumpur, Malaysia; 2 Department of Periodontology and Community Oral Health, Faculty of Dentistry, Universiti Sains Islam Malaysia, Pandan Indah, 55100 Kuala Lumpur, Malaysia; 3 Department of Restorative Dentistry, Faculty of Dentistry, Universiti Kebangsaan Malaysia, Jalan Raja Muda Abdul Aziz, 50300 Kuala Lumpur, Malaysia

**Keywords:** Chewing stick, Siwak, *Salvadora persica*, Gingivitis, Plaque, Gingival recession

## Abstract

**Background:**

Siwak is a chewing stick used as an oral hygiene aid associated with Muslim communities across the globe since more than 1500 years ago. Used either exclusively or in conjunction with a regular toothbrush, there is evidence supporting its clinical effectiveness in plaque control, but adverse effects on periodontal health remains inconclusive.

**Objective:**

This study aims to systematically review the wide range of data and literatures related to siwak practice and its effect on periodontal health.

**Method:**

The review was conducted based on scoping review techniques, searching literature in EBSCOHOST, PubMed, SCOPUS and Google scholar databases using the following search terms: “siwak’ or ‘miswak’ or ‘chewing stick” for intervention, and “periodontium or ‘periodontal’ or ‘periodontal health’ or ‘periodontal disease” for outcome. Articles published between January 1990 to March 2021 and written in English language were included.

**Results:**

A total of 721 articles collected from the search and 21 of them were eligible for the final analysis. Results of this study was described based on clinical and antibacterial reporting of siwak, method of siwak practice and its adverse effect on oral health. Siwak was found effective at removing dental plaque and improving periodontal health over time although its effect on subgingival microbiota was inconclusive. Presence of gingival recession and clinical attachment loss were much more commonly reported in siwak users, attributable to variations in the methods employed for tooth cleaning using the siwak.

**Conclusion:**

There is substantial evidence that the lack of standardised reporting for effective siwak use may have resulted in contradictory findings about its oral hygiene benefits and adverse effects. As such, future work on safe and effective siwak practice is to be advocated among its users.

## Introduction

### Rationale

Siwak is a chewing stick obtained from stem, twig and root of a tree, name Arak (*Salvadora persica*) and used for teeth and oral cleaning. This chewing stick is usually prepared at an average of 1.0 cm in diameter and 15 cm in length to ease its insertion into the mouth and placement on tooth surfaces. Its middle part contains ample phloem and has a spongy texture. After soaking it in water for at least one or two minutes, the stick will become more chewable, hence it becomes easier to remove it while crushing the end portion of the bark, causing it to have a brush-like appearance and ready to use [1, 2].

While siwak had been used by various civilizations [3], for the Arabs it was only during the Islamic period that personal hygiene was further emphasised as part of religious obedience, including the use of siwak as a tool for oral hygiene [4]. As a display of obedience to religious advice, groups of Islamic movement (Jama’ah tabligh) would also constantly have siwak in their pocket [4, 5]. Today, the siwak practice continues and is typically recognised as a cultural identity among Muslim communities.

There are varying reports of siwak users in the developing country and from different regions of Saudi Arabia, Africa, Iran, India and Malaysia [6–11]. The prevalence of adults who use siwak in Cameroon was found to be high (85%) [9], while in Aseer, Saudi Arabia only about half (52.7%) of the adults are reported to use siwak either as a toothbrush replacement, or together with toothbrush. Generally, the adjunctive use of siwak was found to be of personal preference [6] including the majority (73%) of *jamaáh tabligh* congregating at a mosque in Kuala Lumpur, Malaysia [12] where the first mass outbreak of COVID-19 was reported in that country.

At present times where the use of the standard toothbrush is widespread, the cost of siwak may be considered cheaper than the toothbrush especially in countries where its plant source is cultivated locally. Such example is in Uganda, where the two most common plants used as chewing stick are Rhus vulgaris Meikle and Landa trifolia L [13]. The plant sources vary around the world, namely in India, the siwak that is widely used is from Neem (*Azadirachtaindica*); in West Africa the plant source is lime tree (*Citrus aurantafolia*) and orange tree citrus (*Citrus sinensis*); in other parts of Africa it is Senna (*Cassia vennea*), and in the Middle East it is Arak (*Salvadora persica*) [14]. Moreover, while neem is a native plant in India, siwak from its source is also available in Indonesia, Malaysia, Australia, Sri Langka, Burma, Pakistan and Africa [15]. Besides its comparatively low cost to produce, convenient access to the source is another factor promoting the use of siwak.

Apart from these reasons, another main reason people choose siwak was because of religious beliefs [6, 9, 12, 16]. That being acknowledged, only 32.6% of the general Muslim population in Malaysia reported to have had experience of using siwak although almost all study participants were aware that siwak use is a *sunnah* (customary) of the Prophet Muhammad, peace be upon him [17]. Additionally, the method and practice of siwak by the Prophet (PBUH) is generally unknown. Nevertheless, the benefits of siwak on oral health care are acknowledged [17] and for this reason as well as religious beliefs, the use of siwak becomes a. continued practice in muslim communities [16].

### Aims

This study aims to systematically review the literatures on the nature, and extent of siwak use and to identify the gap of knowledge, in relation to the methods of siwak practice. Specifically, this present paper is focused on the clinical benefits of siwak, and its adverse effects to periodontal health in relation to the nature and method of siwak practice.

## Methodology

### Scoping review design

This review process was undertaken based on an established scoping review technique that follows a framework proposed by Joanna Briggs Institute (JBI) and guided by the updated methodology of the Preferred Reporting Items for Systematic Reviews and Meta-Analyses extension for Scoping Reviews (the PRISMA-ScR) [18, 19]. This technique was chosen to enable the exploration of broader research questions and interpret materials from various range of evidences [18, 20]. Data from different types of studies and methodologies that are relevant to the intervention/concept and outcome/context of the topic were processed.

### Review registration

The review title has been registered with Open Science Framework (OSF registration number: osf-registrations-xzhsk-v1).

### Information sources

The overall review process involved systematic searching and screening of literature, extraction of data from the articles and synthesis of findings. The terms or keywords of “siwak or miswak or chewing stick” correspond to the intervention and “periodontium or periodontal or periodontal health or periodontal disease” for outcome, were used in the search process. These keywords were identified from the initial scoping of the literature and keywords. The search engines in this review were EBSCOHOST (Dentistry and Oral Sciences), PubMed, SCOPUS and Google scholar databases. The search article was filtered for academic journals, human studies, written in English language and published within year 1990 to 2021. Additional relevant publications were found through a manual search of the reference lists of the included studies. The rationale and detail of search string may be found in Table 1.

**Table 1:** Search strategy

Database	Rationale	Search string	Filter
EBSCOHOST (dentistry and oral sciences)	Consist of an extensive collection of essential full-text dentistry journals and many of which are open access	(siwak or miswak or ‘’chewing stick’’) AND ( periodontium or periodontal or ‘’periodontal health’’ or ‘’periodontal disease’’)	Year: 1990–2021, source type: Academic journal, English language
PubMed	Is a primary medical database, allowing for a more permissive search string to include more medical research in the clinical field	(siwak[Title/Abstract] OR miswak[Title/Abstract] OR “chewing stick”[Title/Abstract]) AND (periodontium[Title/Abstract] OR periodontal[Title/Abstract] OR “periodontal health”[Title/Abstract] OR “periodontal disease”[Title/Abstract])	Year 1990–2021, Exclude book and document
SCOPUS	Is a database that consists of comprehensive and rich data in a wide variety of disciplines	( TITLE-ABS-KEY ( siwak OR miswak OR “chewing stick”) AND TITLE-ABS-KEY ( periodontium OR periodontal OR ‘’periodontal AND health’’ OR ‘’periodontal AND disease’’))	Article, Year: 1990–2021, English, Final publication stage, subject area dentistry
Google scholar	Provides an easy way to search for full text or metadata of scholarly literature, across a wide range of publishing formats and disciplines	siwak OR miswak OR periodontium OR periodontal “periodontal disease” “periodontal health’’ ‘’chewing stick’’	Year: 1990–2021

### Selection of sources of evidence and eligibility criteria

The screening was conducted independently by two researchers, which agreed on, i) if abstracts were not present, results and conclusion sections were used to determine relevance, and ii) studies that aimed to assess the oral hygiene practice in their population, were included for the following eligibility assessment, because of the possibility that siwak is an option of oral hygiene tool. Accordingly, studies which reported on the effect of siwak on periodontal health and described how tooth cleaning using siwak was practiced were included for full paper review. The assessment of eligibility was made based on the inclusion and exclusion criteria. Any disagreement between two researchers, were resolved upon consensus meeting with a third researcher. The rationale of inclusion and exclusion criteria was set out in Table 2 and expand on “PCC” mnemonic (population, concept and context) as recommended.

**Table 2:** Inclusion and exclusion criteria

Criterion	Rationale for inclusion and exclusion
Population: Adult	An adult is person who has reached the age of maturity or adulthood [67]. The use of siwak as an alternative oral hygiene tool among adult who are physically and mentally fit, is considered independent behaviour, compared to children. The adults wearing fixed orthodontic appliance are excluded to minimise the effects of plaque-retentive factors and ease toothbrushing [21]
Concept: Effect of siwak on periodontal health Method and practice of siwak	The World Health Organization (WHO) recognises siwak as an alternate oral hygiene, but more research is needed [22]. It is derived from a common plant and comes in different diameter and length, as well as having distinct characteristics from toothbrushes [3]. Thus, the method and siwak practice may differ from the toothbrush and may have favourable and/or adverse effect on oral health, particularly periodontium. According to Shah et al. [23] traditional oral hygiene practises can harm the soft and hard tissues of the mouth
Context: Clinical benefit and adverse effect of siwak	
Study type: Human studies and based on original data analysis	Studies that involved human population provide original data and comprehensive evidence on the clinical effects of intervention, including siwak
Date of publication: From 01 January 1990 to 24 June 2021	Many ancient people were known to use siwak, and the clinical benefit and adverse effect were recognised [3, 4]. Apart from the perceived oral hygiene benefit of siwak, religious beliefs are the primary reason of existing population continues to use it [6, 16]. As a result, the clinical effect of siwak should be observed and reported in academic journals between 1990 to 2021, to secure the recent and dated publication within past 30 years

### Data charting process and data items

Extraction and synthesis of information from the included articles was summarized and presented in tables organized under descriptive, methodological, and thematic categories, correspond to the objective and questions of the review [18, 20]. To make reporting easier, a charting table was created during the protocol stage to summarise and record the information according to description of author, reference, and findings. The table was updated throughout the review stage. The data extraction using charting form was piloted with two researchers on three studies. The researchers refined the data to ensure that they were aligned with the research question. A critical evaluation is made on literatures associated with the effects of siwak practice on periodontal health among adults and aimed to answer the following review questions:

1. What are the clinical benefits of siwak on adult oral health?

2. What are the common methods and practices of siwak?

3. Does method and siwak practice contribute to the adverse effect to periodontal health?

The potential data are considered based on the method of siwak brushing and practice and its effect on periodontal health. Although it physical features is different with conventional toothbrush, the users apply similar technique of toothbrushing [3, 16]. Because of that, the efficiency of siwak in removal of plaque, whether supragingival or subgingival may be questioned. Alternatively, varying frequency of siwak use was applied throughout the day [16]. The outcome of toothbrushing also depending on frequency and duration of toothbrushing. However, excessive tooth brushing might cause soft tissue and hard tissue injury such as gingival recession, abrasion and tooth wear [24].

## Result

### Synthesis of result

**Fig. 1:**
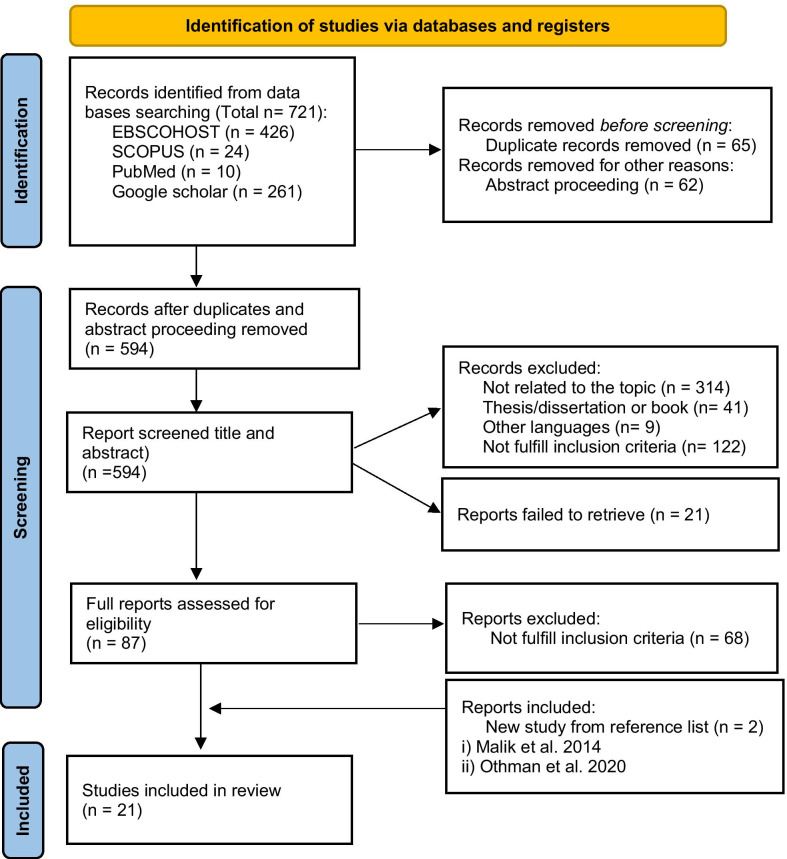
Flow-chart of selection of studies for the review

A total of 721 articles were identified from the initial search, then 65 replicates and 62 abstract conferences were removed and lastly 594 were screened based on the titles and abstracts with reference to the inclusion and exclusion criteria as illustrated in Table 2. Following the screening process, more than half of the articles were further excluded due to the following factors: irrelevant to the topic (314), did not fulfil the inclusion intervention or outcome (122), article published in other languages (9) and were in the form of thesis or dissertation (41). At the end of the screening, a total of 87 articles were deemed relevant, plus two additional articles which were hand-searched from reference lists of included studies. Finally, a total of 21 articles were included for this report and they comprised of original studies involving adult populations from nine randomised-controlled trial (RCT); ten cross-sectional studies; and two case reports. The results from this multi-stage systematic sorting process were summarised and presented in Fig. 1.

The goal of this scoping review was to gather the findings and present an overview of the research rather than to assess the quality of the individual studies. As a result, our overall assessment was narrative rather than quantitative. The descriptive result was summarised with regards to the effect of siwak on oral health and presented according to the following themes: (1) clinical effect of siwak on periodontal health, (2) antibacterial effect of siwak, and (3) method of siwak practice and adverse effect.

### Clinical effects of siwak on periodontal health

Descriptions of studies reporting clinical effects of siwak on oral health are summarised in Table 3. Based on the observational, analytical and cross-sectional studies, the effect of siwak on plaque removal and improvement of gingival health was comparable to that of using toothbrush, whether its use was exclusive or adjunctively [26]. The mean plaque score of siwak user was comparable to toothbrush users, even if used exclusively [27]. Moreover, the mean probing pocket depth (PPD) and gingivitis score were found lower in siwak users compared to toothbrush users [28]. Although PPD and clinical attachment loss (CAL) were comparable to toothbrush, the mean plaque score and bleeding score were significant lower in siwak user, [29]. Despite the lower number of sextants with gingival bleeding and probing pocket depth ≥ 4 mm, there were higher sites of CAL ≥ 4 mm noted in siwak users [30]. Additionally, the mean PPD and CAL were greater, and more sites with gingival recession (REC), when siwak used adjunctively [26, 31]. Inversely, recent works by Ramadan & Alshenqiti discovered significant lower means of PPD, CAL and plaque score, in similar siwak application, compared to toothbrush [32].

**Table 3:** Clinical effect of siwak on periodontal health: cross sectional study

References	Study groups (n = sample size)	Siwak application	Periodontal parameter	Finding
Eid et al. [26]	TB (n = 94) S (n = 68) S&TB (n = 74)	Exclusive Adjunctive	PI, GI, PPD, CAL, REC	Mean plaque score and gingivitis score was comparable with TB
Khawaja et. al. [33]	TB (n = 30) S (n = 30)	Exclusive	PI, GI	
Batwa et al. [27]	TB (n = 29) S (n = 17)	Exclusive	PI	Mean plaque score was comparable with TB
Shetty et al. [28]	TB (n = 216) S (n = 144) S&TB (n = 168)	Exclusive	OHI-S, GI, PPD, REC	Mean plaque scores comparable with TB Significant lower gingivitis score and probing pocket depth Significant higher gingival recession
Al-Sinaidi [29]	TB (n = 74) S (n = 113)	Exclusive	PI, BOP, PPD, CAL	Significant lower mean plaque score and bleeding score Mean PPD and CAL were comparable with TB
Darout et al. [30]	TB (n = 104) S (n = 109)	Exclusive	CPI: BOP, PPD, Calculus	Lower no. sextant of gingival bleeding, probing pocket depth ≥ 4 mm Higher number of sextants with ≥ 4 mm CAL
Eid et. al [26, 31]	TB (n = 94) S (n = 68) S&TB (n = 74)	Adjunctive	PI, GI, PPD, CAL, REC	Significant higher mean PPD and CAL Higher percentage sites with REC
Ramadan et al. [32]	TB (n = 78) S (n = 36) S&TB (n = 36)	Adjunctive	PI, PPD, CAL	Lower mean plaque score, PPD and CAL, than TB

OHI-S, simplified oral hygiene index; PI, plaque index; GI, gingivitis index; BOP, bleeding on probing; PPD, probing pocket depth; CAL, clinical attachment loss; REC, gingival recession; S, siwak; TB, toothbrush

According to clinical studies employing cross-over randomised controlled trials (Table 4), significant reductions of plaque and gingival scores were observed among siwak users compared to the toothbrush users [34, 35]. However, its effect on improvement of gingival health was comparable with that of toothbrushing [36]. Furthermore, Bhambal et. al. found that siwak was equally effective to reduce plaque as well as improving gingival health [37]. It was observed that there were significantly greater reductions of plaque score and gingivitis, when siwak was used as an adjunct to the toothbrush [38–40].

**Table 4:** Clinical effect of siwak on periodontal health: Randomised controlled trial RCT)

References	Study design	Study group (n = sample size)	Siwak application	Periodontal parameter	Finding
Gazi et al. [41]	Cross over	TB (n = 10) S (n = 10)	Exclusive	PI, GI	Significantly reduced mean plaque and gingivitis scores
Al-Otaibi et al. [25]	Cross over	TB (n = 15) S (n = 15)	Exclusive	PI, Plaque-stained surface, GI	
Baeshen et al. [35]	Cross over	TB (n = 15) S (n = 15)	Exclusive	PI	Percentage of plaque was comparable with TB
Bhambal et al. [37]	Cross over	TB (n = 30) S (n = 30)	Exclusive	PI, GI	Reduced mean plaque and gingivitis scores, but no significant difference with TB
Malik et al. [36]	Parallel	TB (n = 25) S (n = 25)	Exclusive	PI, GI	Significantly reduced plaque Comparable with TB in improving gingival health
Patel et al. [38]	Parallel	TB (n = 10) S (n = 10) S & TB (n = 10)	Adjunctive	PI, Plaque-stained surface, GI	Significantly greater reduction of plaque and gingivitis score
Othman et al. [40]	Parallel	TB (n = 10) S (n = 10) S & TB (n = 10)	Adjunctive	PI, GI	Significantly greater reduction of plaque and gingivitis score
Rifaey et al. [39]	Cross over	TB (n = 10) S & TB(n = 10)	Adjunctive	PI, GI, BOP	Significantly greater reduction of plaque and gingivitis score

PI, plaque index; GI, gingivitis index; BOP, bleeding on probing; TB, toothbrush; S, siwak

### Antibacterial effect of siwak

There were four studies which reported the antibacterial effects of siwak (Table 5) from their randomised controlled trials which compared exclusive use of siwak with toothbrushing and its effect on the quantity of subgingival microbiota [39, 42–44]. There was significantly higher quantity of *Aggregatibacter actinomycetemcomitans, Veillonella parvula, Actinomyces israelii, Capnocytophaga gingivalis* and *Streptococcus intermedius* in the siwak groups [42] compared to toothbrushing groups. However, a greater reduction in the number of *A. actinomyctemcomitans* was found in the subgingival plaque of siwak user compared to toothbrush [34]. In contrast, there was no significant difference of *A. actinomycetemcomitans* and *Streptococcus Mutan,* in supragingival plaque, between these groups [39].

**Table 5:** Antibacterial effect of siwak

References	Study type	Siwak application	Sample collection	Microbiological assessment	Finding
Darout et al. [44] and Darout and Skaug [42]	Cross sectional	Exclusive	Subgingival plaque	Whole DNA probe and checkboard DNA-DNA hybridisation	Significantly higher prevalence of *Aggregatibacter actinomycetemcomitans, Veillonella parvula, Actinomyces israelii, Capnocytophaga gingivalis* and *Streptococcus intermedius*
Al-Otaibi et al. [34]	RCT	Exclusive	Subgingival plaque	Whole DNA probe and checkboard DNA-DNA hybridisation	Reduced number of *A. actinomyctemcomitans*
Rifaey et al. [39]	RCT	Adjunctive	Supragingival plaque	Quantitative real-time PCR	No significant difference of *A. actinomycetemcomitans* and *Streptococcus Mutan*

RCT, randomised controlled trial; DNA, deoxyribonucleic acid

### Method of siwak practice and adverse effects

Majority of the studies included in this review did not report on the method or technique of siwak used as a tooth cleaning tool and had no description on the frequency and duration of the daily siwak practice. Nonetheless, in studies that mentioned tooth cleaning methods, descriptions such as siwak being applied in either vertical direction or combination of horizontal directions were frequently cited, as shown in Table 6 [26, 31, 35, 45].

**Table 6:** Method of siwak practice and adverse effect

References	Study type	Method	Practice (frequency and duration)	Adverse effect
Eid et al. [26, 31]	Cross sectional	Vertical	1–5 times/day	Higher mean CAL and REC on midbuccal surface
Darout et al. [30]		Not reported	At least once daily	Higher number of sextants with ≥ 4 mm CAL
Baeshen et al. [35]	RCT	Vertical and horizontal	2 times/day, 5 min	Traumatic lesion on gingival tissue
Al-Otaibi et al. [25, 34]		Not reported	5 times/day	Not reported
Bhambal et al. [37]		Not reported	2 times/day	Not reported
Patel et al. [38]		Not reported	3 times/day	Not reported
Malik et al. [36]		Not reported	2 times/day, 2–5 min	Not reported
Othman et al. [40]		Not reported	2 times/day, at least 2 min	Not reported
Rifaey et al. [39]		Not reported	2 times/day	Not reported
Karia and Kelleher [45]	Case report	Scrubbing motion on every tooth surface, horizontal on buccal and vertical on lingual	Not reported	Severe cervical tooth surface loss (buccal and lingual) and generalised gingival recession
Saleh et al. [5]		Not reported	Not reported	Gingival recession

RCT, randomised controlled trial

Severe gingival recession and tooth surface loss was discovered on the buccal and lingual teeth of a woman that used siwak for toothbrushing in vertical and horizontal directions [45]. The same method of tooth cleaning was applied in a clinical trial and signs of traumatic lesions were reported on gingival tissue [35]. Without reporting the method of siwak use and practice, Saleh et al. discovered gingival recession on labial surface of anterior teeth of 65% of *jamaah tabligh* [5].

The description of siwak practice is made based on the frequency and duration of its daily use as summarised and listed in Table 6. The frequency of siwak use was stated within the range of one to five times in a day, and duration of toothbrushing last was at least two minutes. The frequency of five times daily and brushing in vertical direction was practised by siwak users, and resulted in clinical attachment loss and gingival recession [31]. Another observation among siwak users showed that although the frequency of use was at least once daily, there were more sites with clinical attachment loss of at least 4 mm [30]. The frequency of siwak use in the design of the clinical trials was between two to five times [25, 34, 37–39]. Other clinical trials reported the duration of siwak use to be between two to five minutes [35, 36, 40].

## Discussion

The *Salvadora persica* tree is considered as the main source of siwak in many countries. It is commonly found in Algeria, Egypt, India, Nigeria, Pakistan, Saudi Arabia, Sri Lanka, Uganda and Zimbabwe [46]. With regards to research done on siwak, the same source of siwak is also utilised in most in-vivo and in-vitro studies. Siwak from *S.persica* tree is the most common use for oral hygiene. The siwak practice started at young age population in countries such as India, Sudan, Tanzania, Saudi Arabia and Yemen [8, 10, 47–50]. This early exposure to siwak use explains why the prevalence of siwak use increases in young adult and highest in elderly as its use is likely to have become a habit from a young age and persist till old [43, 51, 52].

Our review found that the main reasons of choosing siwak as an oral hygiene tool is likely to be due to religous beliefs [6, 9, 16, 43]. In addition, the specific features of siwak in its natural form had been claimed to ease its application on the teeth; specifically its small head may facilitate better access to the posterior teeth. Moreover, the availability of the source of supply which is direct from a tree contributes to its low cost. All these factors promote the use of siwak for oral hygiene care [9]. Almost 85% of users reported to feel fresh and whiter teeth after the use of siwak [6]. Other users noticed the absence of gum bleeding and improved oral health, and perceived oral health benefit of siwak use, and these factors has influenced them to choose siwak over toothbrush [16].

### The clinical benefits of siwak on periodontal health

The oral hygiene and gingival health of siwak users were found to be comparable to tooth brush users [26, 31, 33, 37]. Moreover, significant antiplaque and antigingivitis effects were discovered in the randomised controlled clinical trials and analysed in this review [25, 34–36, 41]. Equally important is the finding that significantly greater reductions of plaque and gingivitis scores were observed when siwak was used as an adjunct to the toothbrush [38, 39]. These observations indicate that siwak was either equally effective as toothbrush for mechanical plaque removal or in some studies its use was seen to be superior. These positive benefits support the World Health Organisation (WHO) recommendation on the use of siwak as an alternative measure to the toothbrush for oral hygiene care [22]. Furthermore, siwak exhibits a similar impact as the use of stannous fluoride in the reduction of dental plaque and gingivitis [53].

Gingivitis is an early stage of periodontal disease, and if not treated it may progress to periodontitis causing destruction to the tooth supporting structure and at worst will result in tooth loss. Moreover, there is strong evidence that associate periodontal disease with systemic disease, such as diabetes. This condition may complicate the treatment, increase financial burden and have a negative impact on quality of life [54]. Systematic maintenance of bacteria plaque removal is crucial to prevent reinfection and further bone loss, suggesting long-term dependency on dental visits [55].

It is now known that for the success of periodontal care, it is best that management is personalised according to their genomic and clinical findings and therefore oral hygiene care is still the cornerstone of periodontal disease prevention. As such, self-performed mechanical plaque removal (SPMPR) is important to improve the periodontal health and prevent primary periodontitis (Needleman et al., 2015). The mechanical effect of siwak seems proven to distrupt the bacterial plaque and improved the periodontal health as shown by the lower gingivitis score, probing pocket depth and fewer sites of pocket ≥ 4 mm, found in siwak users [28, 30]. However, the effect of siwak on subgingival plaque microbiota was found to be inconsistent.While higher quantities of *Aggregatibacter actinomycetemcomitans, Veillonella parvula, Actinomyces israelii, Capnocytophaga gingivalis* and *Streptococcus intermedius* were reported in siwak users [44], *A. actinomyctemcomitans* quantities were observed to be lower compared to toothbrush users [34]. Yet recently Rifaey et al. reported that there was no significant difference of *A. actinomycetemcomitans* between siwak and toothbrush user [39].

These observations contradict findings from an in-vitro study which recorded benzyl isothiocyanate (BITC) as the major antibacterial compound of *S.persica* extract that is responsible to inhibit gram negative bacteria, including *A. actinomycetemcomitans, Porphyromonas gingivalis* and *Streptococcus Mutan* [56]. *P. gingivalis* was the most sensitive to BITC and essential oil, compared to *A. actinomycetemcomitans* and *Haemophilus influenza* [57]. Antibacterial activity against gram negative bacteria was highly evidenced in water-based preparation of *S.persica* extract [58]. Furthermore, periodontal pathogens (*Streptococcus mutans, Prevotella intermedia & Peptostreptococcus and Candida albicans*) were significantly sensitive to both water and alcohol extractions [59]. There seems to be a discrepancy in the effects of siwak on the subgingival microbiota between in-vitro and in-vivo study. The reason could be due to unstandardised protocol in preparation of specimen. For instance, there was unmeasured quality of the freshly cut siwak used for everyday toothbrushing in the clinical trials. Instead, the essential oil used in laboratory tests was extracted from the fresh cut of S. persica and standardised to contain the highest concentration of antibacterial compound and produce optimum effects.

The differences in the frequency of siwak practice in the clinical trials may contribute to the inconsistent reports related to the antibacterial effects of siwak [25, 34, 37–39]. According to Albabtain et al. (2018), antibacterial compounds in the siwak brushes reduced significantly from baseline, after being used more than once. The reduction of the same antibacterial compounds was also observed in the saliva, and the compound disappeared after ten minutes [57]. There were several clinical trials that applied the extended duration of siwak brushing than conventional toothbrush practice and this measure should give more chance of getting the benefit from released chemical compounds [35, 36].

The quantified microbiota plaque in those studies were collected from subgingival areas of the study participants [34, 39, 44]. These subgingival areas are naturally formed, when the gingival margin is sealed at the cervical of tooth (cementoenamel junction) through junctional epithelium, creating a narrow space between tooth surface [60]. Such anatomical arrangement may limit the mechanical action of siwak and as a result, subgingival plaque remains undisturbed. The architecture of established multispecies community of oral biofilm make them tolerant to antibacterial compound [61], unless an appropriate method, such as by using siwak or any other toothcleaning method is able to remove the subgingival plaque within these areas.

### The adverse effect of siwak practice on periodontal health

Most of the reported clinical trials did not describe the details of siwak practice, either concerning the technique of tooth cleaning, the duration or the time taken in using the siwak [34, 36, 38, 39]. The lack of information in these studies raises concerns about their reproducibility and may cause any oral health benefits discovered from their research to be deemed as be less meaningful.

Eid and co-workers noted of significant gingival recessions on the labial surface of premolars and central incisors of siwak users [31]. In addition, Baeshen and co-workers also found signs of traumatised tissues on the gingiva. The horizontal toothbrushing is common and easiest to apply, and according to Bergström and co-workers, this method is highly associated with gingival recession and abrasion [62]. The most common method of siwak use observed among users was vertical and/or horizontal directions [26, 31, 35, 45].

In spite of reductions in plaque, gingivitis and periodontal pocket depth among siwak users, there appears to be more sextants associated with clinical attachment loss [30]. In a case study, one patient presented with severe tooth surface loss on buccal and lingual surfaces, as well as generalised recession, but there was absence of any periodontal pocket. Investigations to locate any etiologic factor prior to restorative treatment suggested that siwak practice may be a probable cause. The patient used the average sized siwak in scrubbing motion on all tooth surfaces, horizontal on buccal and vertical on lingual [45]. Incorrect method of brushing and hard texture of siwak fibers were suspected as the cause of the gingival recession, tooth abrasion and signs of oral soft tissue trauma in long term siwak users [5]. This might explain the higher incidence of gingival recession in populations that use traditional oral hygiene tools such as siwak [23]. It is undeniable that hard bristle contributes to the occurrence gingival recession [63]. Nevertheless, the most important toothbrushing factors that have been associated with the development and progression of gingival recession are frequency and method of brushing [64].

The frequency of toothbrushing in siwak user was between one to five times per day [26, 31], although siwak use may be expected to be at least five times daily or more, based on Islamic religious advice. Siwak is also reported to be frequently used on special days like Friday and during religious special events [16]. The extreme frequency and lengthy oral hygiene practice are secondary influence factors for the development and progression of gingival recession [64]. Recently, a survey among a small group of Muslim siwak users while visited a Mosque in Kuala Lumpur, reported that most of method and siwak practice was according to religious advice [7]. However, the method of siwak practice by Prophet (saw) was not clearly understood in most of Malaysian population [65]. Thus, the instruction on proper method of siwak practice is required with consideration of optimum clinical effectiveness and safety on the oral soft tissues.

Integration of oral hygiene instruction with self-performed mechanical plaque removal is expected to prevent soft tissue trauma and achieve high standard of daily plaque control [24, 66]. Appropriate oral hygiene education should include knowledge on proper method of siwak practice for existing siwak users and communities of siwak users from different cultures and beliefs. Even among Asian dental educators, knowledge and awareness towards siwak practice is still lacking and this needs to be addressed if proper use of siwak is to be advocated [64].

## Conclusion

This scoping review provides description of the clinical benefit and adverse effect of siwak on periodontal health, based on the evidence of observation and examination among adults. Unmistakeably, evidence-based instructions on safe and effective method and practice of siwak as an oral hygiene tool is still lacking in the literature. The user continues to practice according to their beliefs and there is a risk that improper use may damage oral tissues. To quantitatively measure the effectiveness of intervention and to qualify each included study, systematic reviews and meta-analyses are now required. Furthermore, it is important for future research on oral hygiene instructions for siwak use are developed based on an integrative approach between scientific evidence and cultural considerations.

## Data Availability

The datasets used and/or analysed used in this study are available from the corresponding author on reasonable request.

## Abbreviations

PI: Plaque Index
GI: Gingivitis Index
BOP: Bleeding on probing
TB: Toothbrush
S: Siwak
OHI-S: Simplified Oral Hygiene Index
PPD: Probing pocket depth
CAL: Clinical attachment loss
REC: Gingival recession
RCT: Randomised controlled trial
DNA: Deoxyribonucleic acid
WHO: World Health Organisation
SPMPR: Self-performed mechanical plaque removal
